# Effect of maternal praziquantel treatment for *Schistosoma japonicum* infection on the offspring susceptibility and immunologic response to infection at age six, a cohort study

**DOI:** 10.1371/journal.pntd.0009328

**Published:** 2021-04-16

**Authors:** Susannah Colt, Blanca Jarilla, Palmera Baltazar, Veronica Tallo, Luz P. Acosta, Hannah W. Wu, Christopher V. Barry, Jonathan D. Kurtis, Remigio M. Olveda, Jennifer F. Friedman, Mario A. Jiz

**Affiliations:** 1 Center for International Health Research, Rhode Island Hospital, The Warren Alpert Medical School of Brown University, Providence, Rhode Island, United States of America; 2 Department of Pediatrics, The Warren Alpert Medical School of Brown University, Providence, Rhode Island, United States of America; 3 Research Institute for Tropical Medicine, Manila, The Philippines; 4 Remedios Trinidad Romualdez Hospital, Tacloban City, Leyte, The Philippines; 5 Department of Pathology and Laboratory Medicine, The Warren Alpert Medical School of Brown University, Providence, Rhode Island, United States of America; Uniformed Services University of the Health Sciences, UNITED STATES

## Abstract

In areas endemic to schistosomiasis, fetal exposure to schistosome antigens prime the offspring before potential natural infection. Praziquantel (PZQ) treatment for *Schistosoma japonicum* infection in pregnant women has been demonstrated to be safe and effective. Our objectives were to evaluate whether maternal PZQ treatment modifies the process of *in utero* sensitization to schistosome antigens potentially impacting later risk of infection, as well as immune response to *S*. *japonicum*. We enrolled 295 children at age six, born to mothers with *S*. *japonicum* infection who participated in a randomized control trial of PZQ versus placebo given at 12–16 weeks gestation in Leyte, The Philippines. At enrollment, we assessed and treated current *S*. *japonicum* infection and measured serum cytokines. During a follow-up visit four weeks later, we assessed peripheral blood mononuclear cell (PBMC) cytokine production in response to soluble worm antigen preparation (SWAP) or soluble egg antigen (SEA). Associations between maternal treatment group and the child’s *S*. *japonicum* infection status and immunologic responses were determined using multivariate linear regression analysis. PZQ treatment during pregnancy did not impact the prevalence (*P =* 0.12) or intensity (*P =* 0.59) of natural *S*. *japonicum* infection among children at age six. Among children with infection at enrollment (12.5%) there were no significant serum cytokine concentration differences between maternal treatment groups. Among children with infection at enrollment, IL-1 production by PBMCs stimulated with SEA was higher (*P =* 0.03) in the maternal PZQ group compared to placebo. Among children without infection, PBMCs stimulated with SEA produced greater IL-12 (*P =* 0.03) and with SWAP produced less IL-4 (*P =* 0.01) in the maternal PZQ group compared to placebo. Several cytokines produced by PBMCs in response to SWAP and SEA were significantly higher in children with *S*. *japonicum* infection irrespective of maternal treatment: IL-4, IL-5, IL-10, and IL-13. We report that maternal PZQ treatment for *S*. *japonicum* shifted the PBMC immune response to a more inflammatory signature but had no impact on their offspring’s likelihood of infection or serum cytokines at age six, further supporting the safe use of PZQ in pregnant women.

**Trial Registration:** ClinicalTrials.gov NCT00486863.

## Author summary

Children living in areas endemic to schistosomiasis typically do not present signs of acute illness during infection. It is believed that fetal exposure from a pregnant mother with infection primes the offspring and influences the child’s susceptibility and immune response to infection. Praziquantel treatment for *Schistosoma japonicum* infection in pregnant women has been demonstrated to be safe and effective, but the long-term impact on the child’s immunity is not fully understood. This study enrolled 295 children at age six, who were born to mothers who participated in a randomized controlled trial of praziquantel vs placebo for *S*. *japonicum* infection during pregnancy. The children were assessed and treated for *S*. *japonicum* infection and blood was collected to measure immune response markers. We found that praziquantel treatment during pregnancy did not impact the prevalence or intensity of naturally acquired *S*. *japonicum* infection among children at age six. Among the 37 children with *S*. *japonicum* infection, immune response markers did not differ by maternal treatment group. This evidence further supports the safe use of praziquantel for pregnant women.

## Introduction

Schistosomiasis is a parasitic disease that affects more than 140 million people worldwide [1]. Morbidity is of particular concern for children and adolescents, who exhibit greater infection intensity or worm burden [2,3], and for pregnant women, who suffer an increased risk for adverse pregnancy outcomes [4,5]. Untreated infections in children can lead to anemia, malnutrition, and impaired development [6–10]. Treatment with praziquantel (PZQ) is recommended for infected adults and children older than 4 years and is also distributed as part of mass drug administration (MDA) campaigns in endemic areas [11–13]. In a recent randomized placebo-controlled trial conducted in The Philippines, which also serves as the parent trial for the current study, PZQ was shown to be safe and effective for pregnant women infected with *Schistosoma japonicum* [14]. However, the impact of pre-natal PZQ treatment on immunologic responses to *S*. *japonicum* among offspring in early childhood has not been previously evaluated.

The host immune response to schistosomiasis includes both responses that support resistance to infection, as well as responses that drive immunopathology including hepatic fibrosis, anemia of inflammation, undernutrition, and growth faltering. In humans, the pattern of immunopathology experienced varies based on many factors including parasite life cycle stage, primary vs secondary infection, experience with PZQ treatment, genetics, and mode of prior exposure to infection (direct or *in utero*) [15]. A protective immune response against *Schistosoma* spp. can develop over time, typically during early adulthood, and is characterized by a T-helper type 2 (Th2) response mounted against antigens released by dying adult worms [16,17]. Such antigenic responses, characterized by increased T lymphocyte production of interleukins 4 and 5, result when adult worms die naturally over time or following PZQ treatment. Th2-mediated responses also contribute to the formulation of granulomas around schistosome eggs, which can lead to fibrotic lesions over time [18]. Susceptibility to reinfection along with an increased risk for anemia has been associated with a pro-inflammatory response to schistosome egg antigens [8,19,20].

Peri-natal exposure to parasite antigens and maternal antibodies, transferred transplacentally or via breast milk, can result in schistosome-specific immune sensitization that may influence immunity and/or susceptibility to infection [21–23]. In murine models, mice born to dams with *S*. *mansoni* infection experienced reduced morbidity upon primary infection compared to mice born to uninfected dams [24–26]. In human observational studies, infants born to mothers infected with *S*. *mansoni* demonstrated antigen-specific T cell memory and B cell immunity compared to those born to uninfected mothers [27–29]. Human studies examining the longevity of these priming effects beyond infancy report antigen-specific responses in children 7–36 months [30] and 10–14 months of age [31] who had yet to experience primary infection.

Because PZQ treatment during pregnancy has not been widely adopted, there is limited evidence describing how maternal treatment for schistosomiasis modifies the transfer of antigens and antibodies to the fetus and how this influences immune responses and infection risk. While PZQ is safe for pregnant women, with no increased risk for adverse pregnancy outcomes demonstrated [14], long-term effects of pre-natal treatment on pediatric outcomes are not fully understood. Studies from Uganda report that PZQ treatment of *S*. *mansoni*-infected pregnant women had no influence on the immune responses of uninfected children at one year [32] or five years of age [33]. Here we evaluate the impact of pre-natal PZQ treatment of pregnant women infected with *S*. *japonicum* on outcomes of their offspring at age six, including natural infection and intensity, immunologic response to schistosome antigens, and circulating cytokines.

## Methods

### Ethics statement

The study was approved by the ethics review board of the Research Institute of Tropical Medicine in Manila, The Philippines (#2012–021). For each participant, formal written informed consent was obtained from a parent. Children were too young to provide assent.

### Population

**Fig 1:**
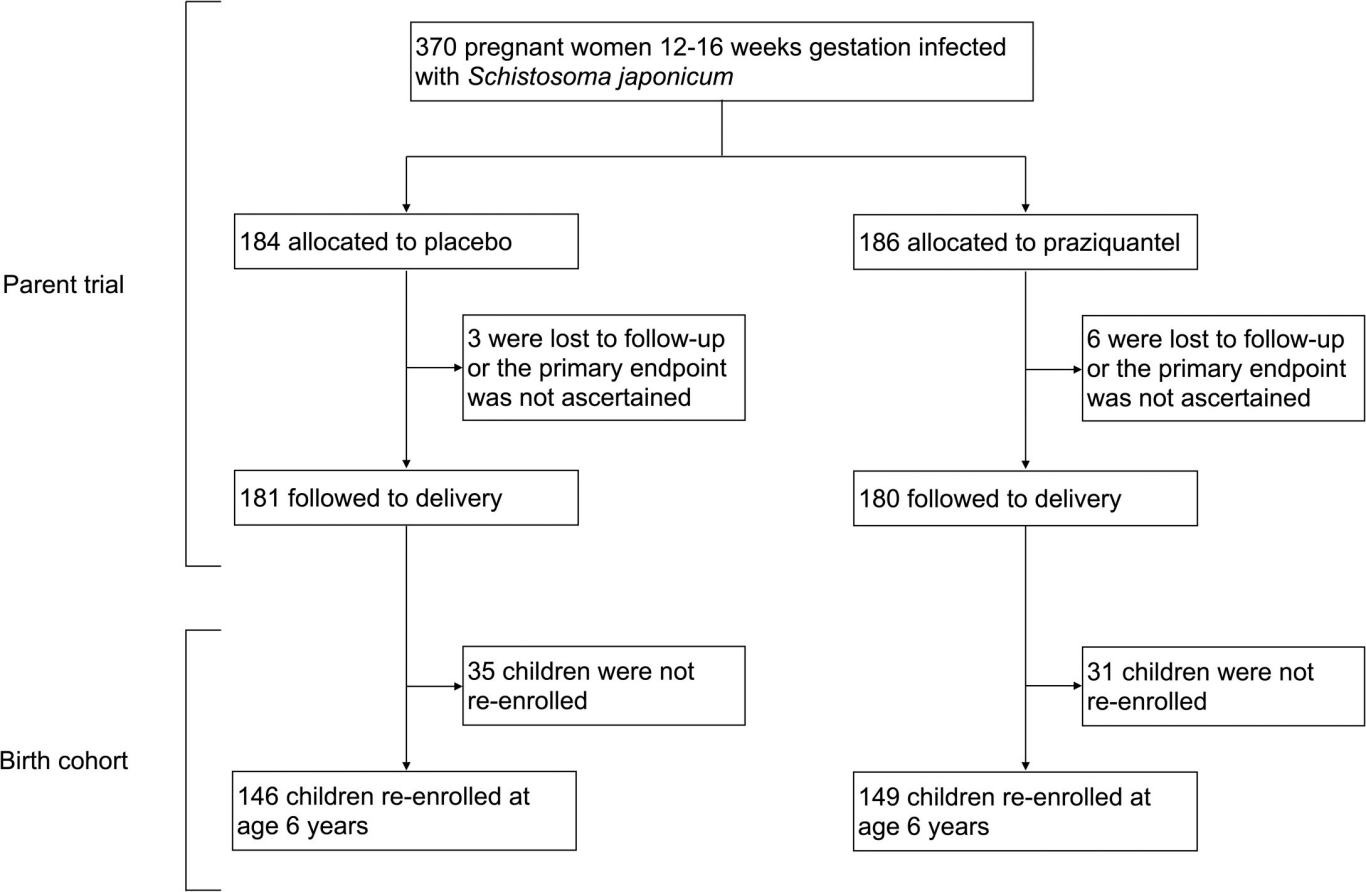
Participants included for analysis.

Participants include children born to pregnant women (N = 361) who were enrolled in a double blind, randomized control trial (RCT) of PZQ versus placebo given at 12–16 weeks gestation in Leyte, The Philippines (ClinicalTrials.gov: NCT00486863) [14], see Fig 1. We invited children to participate when they turned five. Due to disruptions and delays following Hurricane Haiyan, the recruitment age was moved to six years, and remaining children were enrolled at that time. In the present analysis, participants attended two study visits; one at enrollment at age six and one four weeks later. During the enrollment visit, participants were assessed for *S*. *japonicum* infection and if infected, were treated with PZQ (60 mg/kg divided in two doses separated by four hours). Demographic information, anthropometric measures, serum cytokine measures, and soil-transmitted helminth infections were also captured. During the four-week follow-up visit, participants provided a blood sample to capture peripheral blood mononuclear cell (PBMC) responses to schistosome antigens. In the study area, MDA of PZQ is provided at the community level annually as part of preventative chemotherapy campaigns and offered to individuals aged five and older regardless of infection status. Participants could have been eligible to receive MDA in the year prior to enrollment between 2013–2018, however MDA was interrupted from 2013–2014 due to Hurricane Haiyan. We asked participants if they had received treatment in the previous year and only 40 reported receipt through MDA.

### Laboratory measures

#### Helminth infection and intensity

Three stool samples were collected 1–2 days apart as part of the enrollment visit and examined for *S*. *japonicum*, *Ascaris lumbricoides*, *Trichuris trichiura*, *and* hookworm eggs in duplicate using the Kato-Katz thick smear technique. Infection intensity is described as the average number of eggs per gram of stool (EPG) across the three samples, and a positive infection is defined as any eggs identified in any stool sample.

#### Serum cytokines

Following blood collection, serum was separated and stored at -80°C. Serum cytokines collected during the enrollment visit were measured using a custom multiplex assay on a bead-based platform (BioPlex, Bio-Rad) as described previously [6], analyzed at the Research Institute for Tropical Medicine in Manilla, The Philippines. Target analytes include: IFN-γ, IL-1, IL-4, IL-5, IL-6, IL-10, IL-13, Tumor Necrosis Factor alpha (TNF-α), TNF receptor I (TNF-RI), and TNF receptor II (TNF-RII).

#### Peripheral blood mononuclear cell stimulation assays

An ex vivo cytokine stimulation assay was performed using peripheral blood mononuclear cells (PBMCs) collected during the four-week follow-up visit. PBMCs were isolated and stimulated as described [17]. On the same day as blood collection, PBMCs were suspended in RPMI 1640 at a concentration of 2 X 10^6^ cells/mL. In 48-well microtiter plates, 250 μL of antigen stimulant was added to 250 μL of PBMC solution in duplicate wells for a final concentration of 1 X 10^6^ cells/mL. Tissue culture plates were incubated at 37°C and 5% CO_2_, and following five days of incubation, 400 μL of supernatant was harvested for cytokine measurements. Stimulation assay cytokines were measured using a custom multiplex assay on the aforementioned bead-based platform. Target analytes include: IFN-γ, IL-1, IL-2, IL-4, IL-5, IL-6, IL-8, IL-10, IL-12, IL-13, and TNF-α.

Antigen stimulant conditions were as follows: 1) Negative control wells contained added volume of RPMI 1640; 2) Positive control wells included a lipopolysaccharide (LPS) solution (Ultrapure LPS purified from *Escherichia coli* 0111:B4, InvivoGen); 3) *S*. *japonicum* soluble worm antigen preparation (SWAP) was harvested from infected rabbits and prepared as described [34]; and 4) *S*. *japonicum* soluble egg antigen (SEA) was prepared according to standard procedures [35]. The final concentrations for antigen stimulant conditions were LPS, 20μg/mL; SWAP, 25μg/mL; and SEA, 15 μg/mL.

### Statistical analysis

Comparisons of participant characteristics between maternal PZQ treatment groups were assessed by a Wilcoxon rank sum test for continuous variables and a Fisher’s Exact test for categorical variables. All subsequent analyses applied linear regression with natural log-transformed continuous variables, to better approximate normal distributions. Least squared means point estimates were generated to compare antigen-stimulated cytokine response variables between maternal PZQ treatment groups, adjusted for negative control conditions. Given the expectation that *S*. *japonicum* infection at baseline could modify the relationship between maternal treatment and immune responses, results were stratified by infection.

The regression model building process for outcomes related to *S*. *japonicum* infection status in children applied automated stepwise selection with entry and stay thresholds of 0.1 to consider potential confounding variables including maternal treatment group, age, sex, gestational age (GA), small for gestational age (SGA) status, weight-for-age z score (WAZ), height-for-age z-score (HAZ), weight-for-height z-score (WHZ), mother’s socio-economic status (SES), as well as current *Ascaris lumbricoides*, *Trichuris trichiura*, and hookworm infections. P values < 0.05 were considered significant. Statistical analyses were conducted using SAS Studio 3.8 (SAS Institute Inc., Cary, NC).

## Results

### Participant characteristics

Of the 361 pregnant participants included in the PZQ RCT and followed to term, 295 of their children were reached for re-enrollment at age six years and included in the present analysis (Fig 1). For the included participants, 56.3% were male, 22.0% were born SGA, and 50.5% were born to mothers receiving PZQ treatment compared to placebo. Only 37 of the 295 participants born to mothers infected with *S*. *japonicum* during pregnancy (12.5%) were infected with *S*. *japonicum* at age six. Proportions of participants infected with *Ascaris lumbricoides*, *Trichuris trichiura*, or hookworm infection at the time of enrollment were 36.9%, 54.2%, and 8.8% respectively. Comparisons between maternal PZQ treatment group showed no differences in age, sex, GA, SGA status, anthropometry measures, mother’s SES, nutrition biomarkers, anemia status, *S*. *japonicum* infection, other helminth infections, or receipt of MDA within the last year (Table 1). In a separate analysis of the findings presented in Table 1, we excluded 40 participants who reported receipt of PZQ as part of MDA, which also resulted in no differences between maternal PZQ treatment group. Among participants with *S*. *japonicum* infection (N = 37), 67.6% were also infected with *Ascaris lumbricoides*, 62.2% with *Trichuris trichiura*, and 13.5% with hookworm infection. None of these co-infections differed significantly by maternal PZQ treatment group.

**Table 1:** Participant characteristics by maternal treatment group.

	Total (n = 295)	Praziquantel (n = 149)	Placebo (n = 146)	*p* value^1^
**Age (years)**	6.15 (6.08–6.21)	6.14 (6.09–6.21)	6.15 (6.07–6.21)	0.6120
**Sex**				
Male	166 (56.3)	85 (57.1)	81 (55.5)	0.8150
Female	129 (43.7)	64 (42.9)	65 (44.5)	
**Gestational Age (weeks)**	38.7 (38.0–39.4)	38.7 (38.0–39.3)	38.7 (38.0–39.4)	0.8073
**Small for Gestational Age**				
< 10% INTERGROWTH	65 (22.0)	38 (25.5)	27 (18.5)	0.1616
≥ 10% INTERGROWTH	230 (78.0)	111 (74.5)	119 (81.5)	
**Anthropometry**				
Weight-for-Age Z score (WAZ)	-1.9 (-2.6, -1.3)	-2.0 (-2.6, -1.3)	-1.8 (-2.6, -1.2)	0.3992
Height-for-Age Z score (HAZ)	-1.9 (-2.5, -1.3)	-1.9 (-2.5, -1.4)	-1.9 (-2.4, -1.3)	0.8785
Weight-for-Height Z score (WHZ)	-0.8 (-1.5, -0.1)	-0.9 (-1.5, -0.3)	-0.7 (-1.4, -0.1)	0.1985
Underweight (WAZ < -2)	136 (46.1)	75 (50.3)	61 (41.8)	0.1613
Stunting (HAZ < -2)	123 (41.7)	61 (40.9)	62 (42.5)	0.8141
Wasting (WHZ < -2)	36 (12.2)	19 (12.7)	17 (11.6)	0.8594
Body Mass Index (kg/m^2^)	14.5 (13.8–15.1)	14.4 (13.8–15.0)	14.6 (13.9–15.3)	0.2091
**Socioeconomic quartile**				
Q1 (highest)	73 (24.7)	33 (22.1)	40 (27.4)	0.5822
Q2	72 (24.4)	35 (23.5)	37 (25.3)	
Q3	112 (38.0)	59 (39.6)	53 (36.3)	
Q4 (lowest)	38 (12.9)	22 (14.8)	16 (11.0)	
**Nutrition Biomarkers**				
Hemoglobin (g/dL)	12.2 (11.7–13.0)	12.3 (11.7–12.9)	12.2 (11.6–13.2)	0.7858
Ferritin (μg/L)	5.9 (3.5–12.3)	5.8 (3.9–11.1)	5.9 (2.9–13.2)	0.8450
sTfR (mg/L)	1.5 (1.1–2.0)	1.5 (1.1–2.1)	1.5 (1.0–2.0)	0.5636
**Anemia**				
Iron deficiency anemia	47 (16.0)	20 (13.4)	27 (18.5)	0.4481
Non-iron deficiency anemia	3 (1.0)	2 (1.3)	1 (0.7)	
Not anemic	244 (83.0)	127 (85.2)	118 (80.8)	
**Helminth Infection**				
*Schistosoma japonicum*	37 (12.5)	24 (16.1)	13 (8.9)	0.0783
*Ascaris lumbricoides*	109 (36.9)	61 (40.9)	48 (32.9)	0.1814
*Trichuris trichiura*	160 (54.2)	83 (55.7)	77 (52.7)	0.6411
Hookworm	26 (8.8)	12 (8.0)	14 (9.6)	0.6852
***S*. *japonicum* Intensity Category**^**2**^				
Low (1–99 eggs/g stool)	36 (12.2)	23 (15.4)	13 (8.9)	1.0000
Moderate (100–399 eggs/g stool)	0 (0)	0 (0)	0 (0)	
High (≥400 eggs/g stool)	1 (0.3)	1 (0.7)	0 (0)	
***S*. *japonicum* Intensity (eggs/g stool)**^**2**^	6.7 (3.3–16.7)	6.7 (3.3–23.3)	6.7 (3.3–13.3)	0.8823
**Receipt of MDA in the previous year**	40 (13.6)	21 (7.1)	19 (6.4)	0.8655

Values reported in median (IQR) or n (%)

1) P values generated by Wilcoxon rank sum test for continuous variables or Fisher’s Exact test for categorical variables

2) Among cases of *S*. *japonicum* infection, N = 37

MDA, Mass Drug Administration of praziquantel at the community level

### Longitudinal impact of PZQ treatment *in utero*

**Fig 2:**
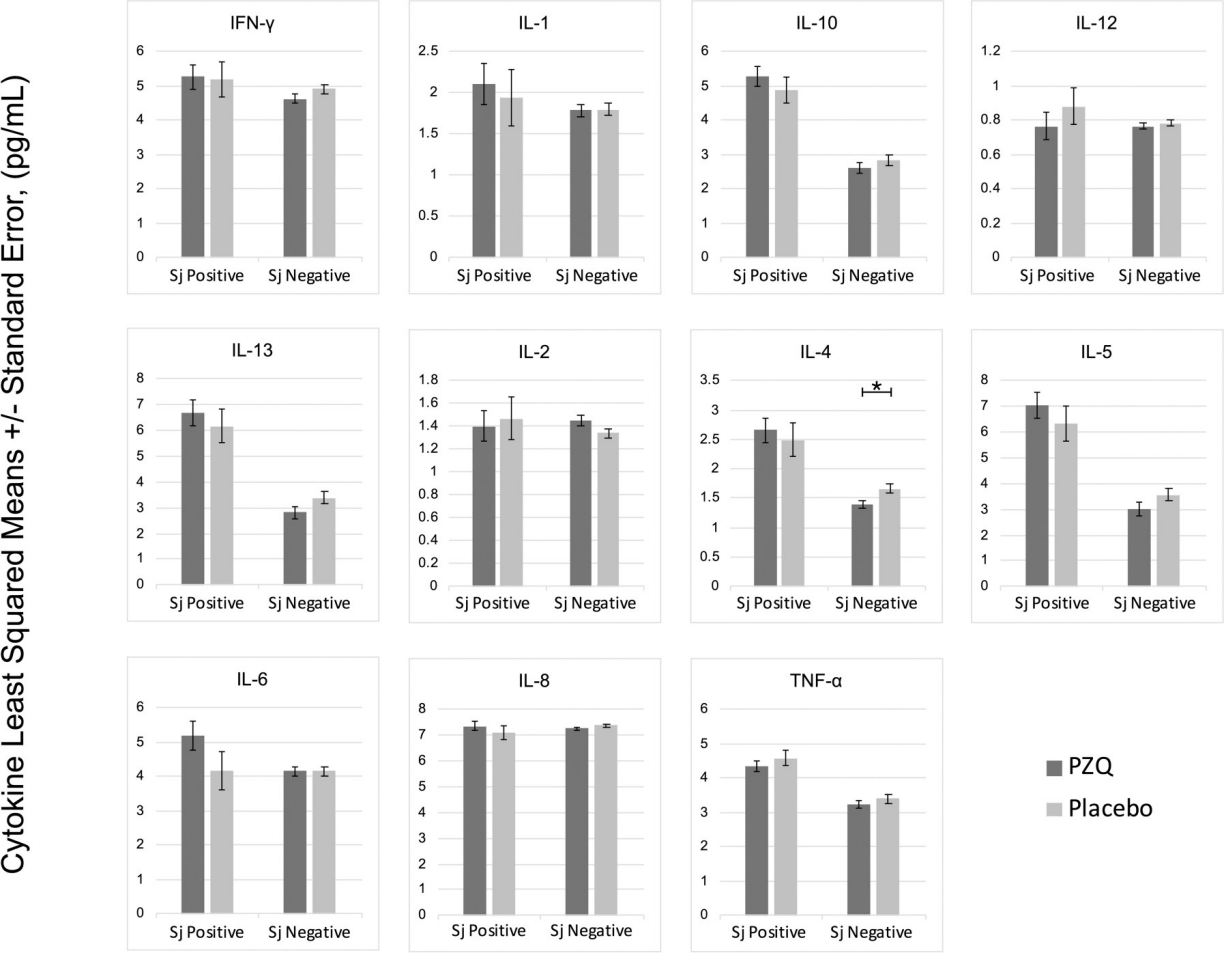
PZQ in utero priming: cytokines stimulated with Soluble Worm Antigen, N = 295. Values are natural log-transformed. Each model adjusted for the antigen stimulation negative control. Asterisks represent significant differences determined by multivariate regression.

**Fig 3:**
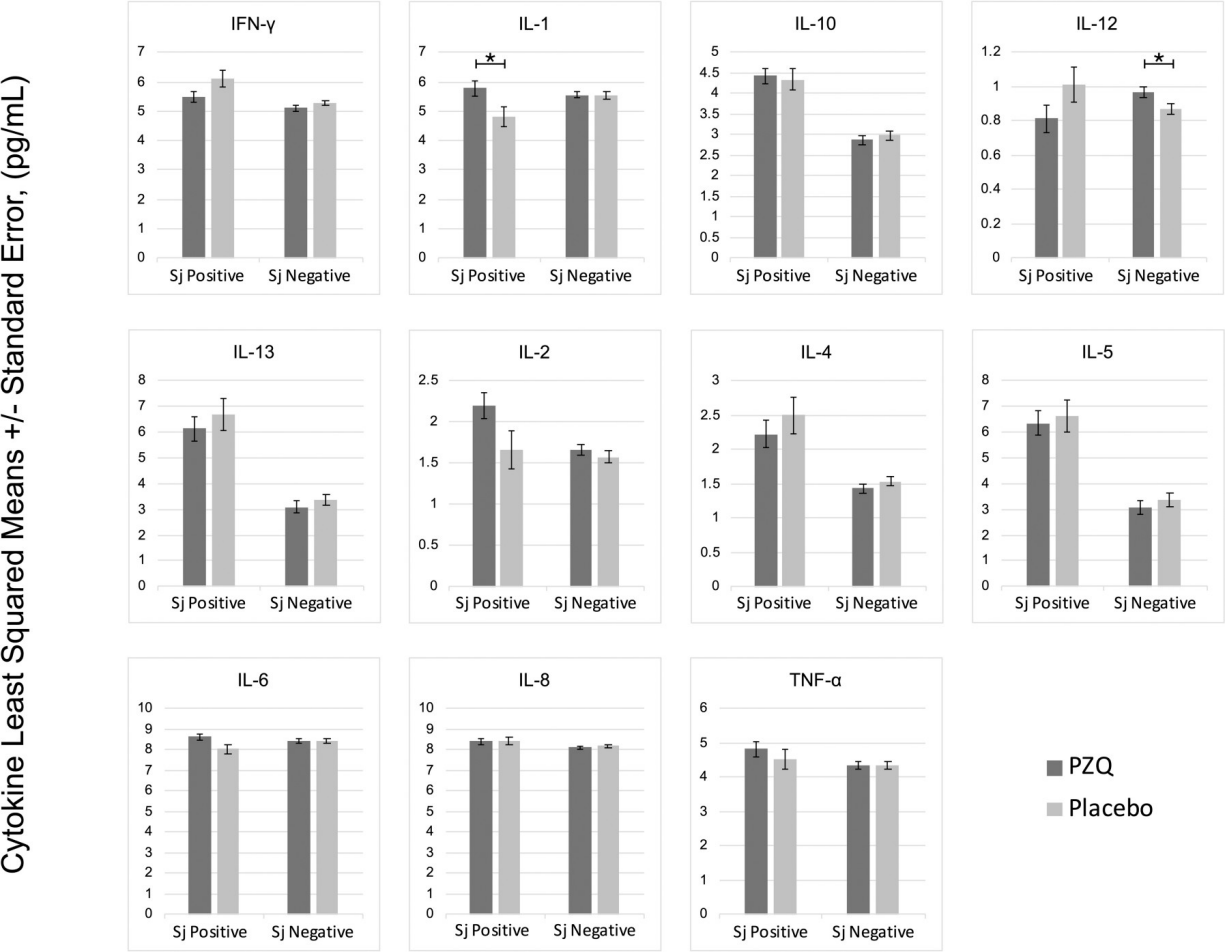
PZQ in utero priming: cytokines stimulated with Soluble Egg Antigen, N = 295. Values are natural log-transformed. Each model adjusted for the antigen stimulation negative control. Asterisks represent significant differences determined by multivariate regression.

**Fig 4:**
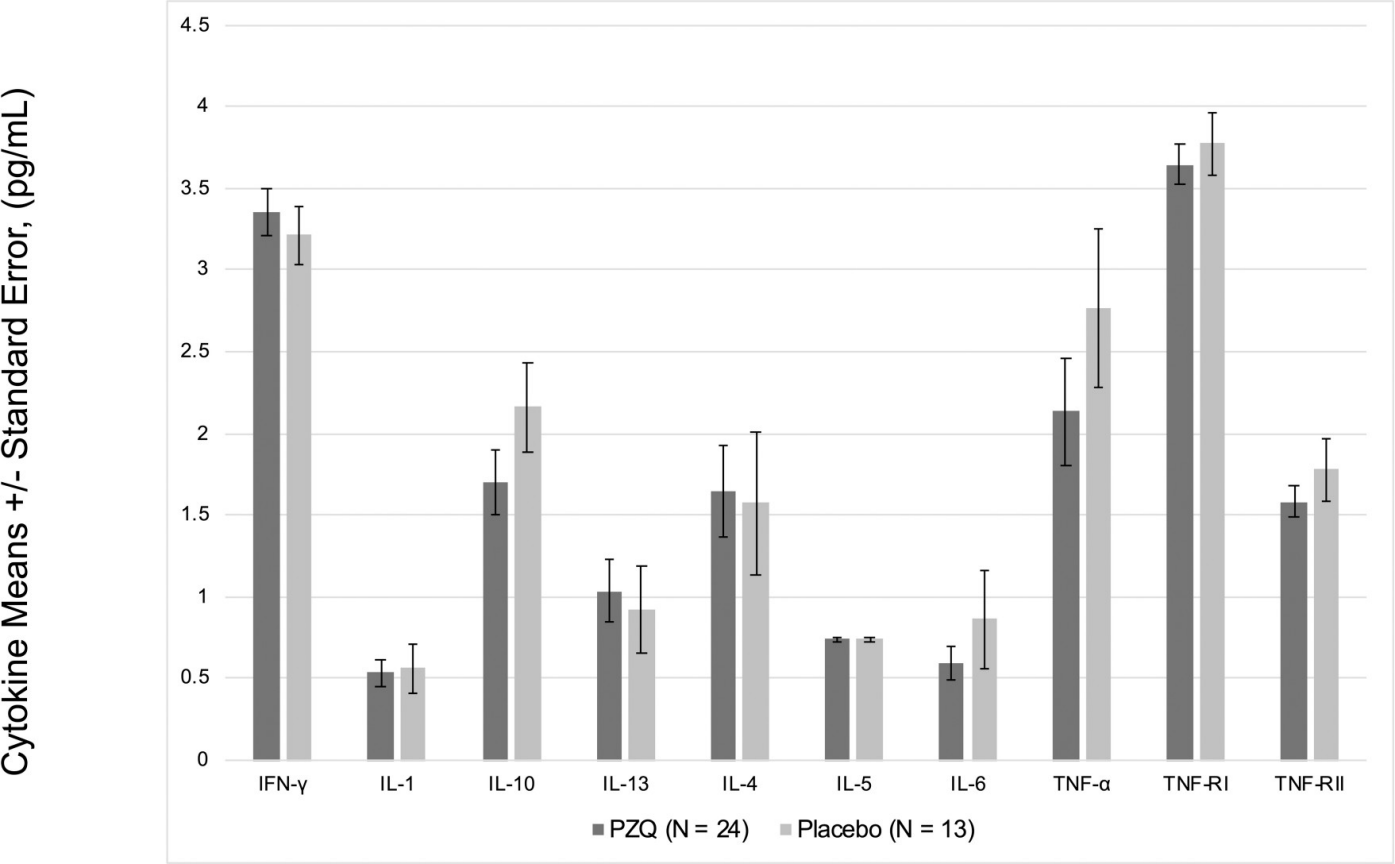
Serum cytokines among children infected with S. japonicum, N = 37. Values are natural log-transformed.

In multivariate regression models adjusting for *Ascaris lumbricoides* infection, maternal PZQ treatment was not associated with *S*. *japonicum* infection (16.1% praziquantel, 8.9% placebo; *P =* 0.12) or *S*. *japonicum* intensity of infection with both groups experiencing a mean EPG of 6.7 among infected children (*P =* 0.59) at age six. We assessed PBMC cytokine responses to SWAP and SEA by maternal PZQ treatment or placebo group, stratified by the child’s *S*. *japonicum* infection status at enrollment, given that several of these responses were significantly higher among children who were infected at enrollment (Figs 2 and 3). For uninfected children, the IL-4 response to SWAP was lower among the maternal PZQ treatment group compared to the placebo group (P = 0.01) and the IL-12 response to SEA was higher among the maternal PZQ treatment group (P = 0.03). For children with *S*. *japonicum* infection at enrollment, the IL-1 response to SEA was higher among the maternal PZQ treatment group than the placebo group (P = 0.03). With regard to serum cytokines, there were no differences between maternal treatment groups in either the total cohort or for children infected with *S*. *japonicum* (Fig 4).

### Immunologic response to acquired *S*. *japonicum* infection

Associations between *S*. *japonicum* infection at enrollment and serum cytokines and antigen-stimulated PBMC responses were assessed in multivariate models and reported in Tables 2–4. Serum TNF-RI was higher in children with *S*. *japonicum* infection (*P =* 0.01; Table 2). None of the associations between *S*. *japonicum* infection and serum cytokines was significantly modified by maternal PZQ treatment group.

**Table 2:** Association between *S*. *japonicum* infection and serum cytokines.

	Median (IQR)			Multivariate linear regression^1^^,^ ^2^		
Cytokines (pg/mL)	Total (n = 295)	Sj Positive (n = 37)	Sj Negative (n = 258)	ß	SE	*p* value
IFN-γ	31.48 (19.36–39.86)	31.48 (19.36–39.86)	31.48 (19.36–39.86)	0.02731	0.14360	0.8493
IL-1	1.83 (1.78–2.21)	1.83 (1.78–1.83)	1.83 (1.78–2.21)	-0.07589	0.09296	0.4149
IL-10	4.07 (2.11–9.63)	7.27 (3.44–13.06)	4.03 (2.11–9.45)	0.25273	0.18040	0.1623
IL-13^3^	2.30 (1.52–6.30)	1.88 (1.40–3.55)	2.68 (1.88–6.30)	-0.15732	0.16783	0.3493
IL-4^3^^,^ ^4^	2.09 (1.88–13.61)	2.09 (2.09–10.83)	2.37 (1.88–13.61)	-0.02494	0.26900	0.9262
IL-5^5^	2.15 (2.07–2.23)	2.15 (1.97–2.15)	2.15 (2.07–2.23)	-0.01152	0.01478	0.4363
IL-6^6^	1.84 (1.71–2.44)	1.84 (1.35–2.44)	1.84 (1.71–2.44)	-0.21880	0.14128	0.1225
TNF-α^4^	3.50 (2.25–39.62)	7.27 (2.42–40.87)	3.50 (2.22–18.90)	0.46209	0.27582	0.0949
TNF-RI	33.43 (19.63–48.39)	42.99 (26.43–58.82)	31.34 (18.45–45.70)	0.39604	0.15183	0.0096
TNF-RII	4.96 (3.70–5.78)	5.42 (4.09–6.65)	4.45 (3.70–5.65)	0.19702	0.13248	0.1381

1) Values are natural log-transformed

2) Stepwise model selection using entry and stay thresholds of 0.1; Included treatment, age, sex, gestational age, small for gestational age, weight-for-age z-score (WAZ), height-for-age z-score (HAZ), weight-for-height z-score (WHZ), socio-economic status, Ascaris infection, Trichuris infection, hookworm infection

3) Ascaris infection retained in multivariate model

4) Height-for-age z-score retained in multivariate model

5) Weight-for-height z-score retained in multivariate model

6) Hookworm infection retained in multivariate model

**Table 3:** Association between *S*. *japonicum* infection and SWAP-stimulated cytokines.

Cytokines (pg/mL)	Median (IQR)			Multivariate Linear Regression^1^^,^^2^		
	Total (n = 295)	Sj Positive (n = 37)	Sj Negative (n = 258)	ß	SE	*p* value
IFN-γ	107.93 (39.60–255.45)	156.38 (56.73–437.40)	95.05 (39.60–255.45)	-0.03662	0.27481	0.8941
IL-1	3.36 (2.48–7.75)	3.46 (2.93–12.65)	3.36 (2.48–7.44)	0.20989	0.15221	0.1690
IL-10^3^^,^ ^4^	9.83 (2.16–141.86)	216.42 (104.07–384.67)	5.19 (2.13–69.46)	2.26796	0.32898	< .0001
IL-12	1.15 (0.81–1.45)	1.18 (0.81–1.36)	1.14 (0.81–1.45)	0.05503	0.04303	0.2019
IL-13^3^^,^ ^4^	6.08 (2.18–423.01)	877.46 (306.76–3554.00)	3.74 (2.14–111.88)	3.19863	0.47960	< .0001
IL-2	2.50 (1.78–4.28)	2.14 (1.39–4.60)	2.50 (1.80–4.28)	0.11322	0.08771	0.1978
IL-4^4^	2.54 (1.93–7.62)	10.59 (6.39–25.24)	2.25 (1.87–4.38)	1.01020	0.16091	< .0001
IL-5	6.36 (2.29–721.94)	1553.00 (442.26–4777.00)	3.82 (2.23–180.56)	3.44684	0.48144	< .0001
IL-6^5^, ^6^	58.50 (9.79–307.00)	164.45 (15.61–315.87)	49.90 (9.08–300.74)	0.50124	0.28228	0.0768
IL-8	2115.00 (810.19–3455.00)	1964.00 (857.47–3441.00)	2143.00 (805.04–3455.00)	0.13867	0.12473	0.2671
TNF-α^4^	34.99 (6.73–92.20)	89.32 (45.91–133.73)	28.68 (5.73–82.76)	1.01245	0.24781	< .0001

1) Values are natural log-transformed; all regression models adjusted for stimulation negative control

2) Stepwise model selection using entry and stay thresholds of 0.1; Included treatment, age, sex, gestational age, small for gestational age, weight-for-age z-score (WAZ), height-for-age z-score (HAZ), weight-for-height z-score (WHZ), socio-economic status, Ascaris infection, Trichuris infection, hookworm infection

3) Weight-for-age z-score retained in multivariate model

4) Ascaris infection retained in multivariate model

5) Hookworm infection retained in multivariate model

6) Height-for-age z-score retained in multivariate model

**Table 4:** Association between *S*. *japonicum* infection and SEA-stimulated cytokines.

Cytokines (pg/mL)	Median (IQR)			Multivariate Linear Regression^1^^,^ ^2^		
	Total (n = 295)	Sj Positive (n = 37)	Sj Negative (n = 258)	ß	SE	*p* value
IFN-γ	155.30 (81.54–370.95)	263.64 (126.20–626.45)	147.26 (76.31–341.85)	0.39787	0.19887	0.0464
IL-1	323.37 (126.63–644.88)	245.99 (107.50–559.41)	324.63 (132.29–649.25)	-0.11602	0.23760	0.6257
IL-10	15.72 (6.68–63.39)	64.70 (48.00–208.54)	12.74 (5.49–44.14)	1.45875	0.22735	< .0001
IL-12	1.36 (1.00–1.96)	1.38 (0.97–1.79)	1.36 (1.00–1.98)	0.00539	0.06382	0.9327
IL-13^3^	8.44 (2.76–449.77)	529.25 (184.59–4454.00)	5.12 (2.44–91.50)	3.02200	0.46958	< .0001
IL-2	3.58 (1.82–6.08)	5.37 (2.95–12.11)	3.19 (1.82–5.93)	0.46831	0.14549	0.0014
IL-4	2.37 (1.93–6.36)	7.37 (4.28–16.33)	2.25 (1.87–4.06)	0.80134	0.14121	< .0001
IL-5^3^	7.29 (2.29–413.38)	672.24 (185.81–4602.50)	3.79 (2.23–129.22)	3.31255	0.48590	< .0001
IL-6	5806.00 (4229.00–7890.00)	6048.50 (3982.50–7890.00)	5793.00 (4251.00–7890.00)	-0.01803	0.18768	0.9236
IL-8^4^	3444.00 (3103.00–4444.00)	3785.00 (3215.00–9430.00)	3444.00 (2956.00–4050.00)	0.21461	0.13812	0.1214
TNF-α	91.13 (39.97–207.73)	132.12 (52.44–284.83)	86.62 (39.37–195.17)	0.40005	0.22301	0.0739

1) Values are natural log-transformed; all regression models adjusted for stimulation negative control

2) Stepwise model selection using entry and stay thresholds of 0.1; Included treatment, age, sex, gestational age, small for gestational age, weight-for-age z-score (WAZ), height-for-age z-score (HAZ), weight-for-height z-score (WHZ), socio-economic status, Ascaris infection, Trichuris infection, hookworm infection

3) Weight-for-age z-score retained in multivariate model

4) Height-for-age z-score retained in multivariate model

For PBMCs stimulated with SWAP, there were positive associations between *S*. *japonicum* infection and IL-4 (*P* < 0.0001), IL-5 (*P* < 0.0001), IL-10 (*P* < 0.0001), IL-13 (*P* < 0.0001), and TNF-α (*P =* 0.0002; Table 3). In response to SEA stimulation, *S*. *japonicum* infection was positively associated with concentrations of IFN-γ (*P =* 0.05), IL-2 (*P =* 0.001), IL-4 (*P* < 0.0001), IL-5 (*P* < 0.0001), IL-10 (*P* < 0.0001), and IL-13 (*P* < 0.0001; Table 4).

## Discussion

*In utero* sensitization to *S*. *japonicum* infection primes the offspring before natural infection. While PZQ treatment in pregnant women has been demonstrated to be safe and effective, it has also been associated with a pro-inflammatory immune response in pregnant women [36]. Given the complex immune responses to schistosomiasis as related to both resistance to infection and immunopathogenesis, modification of these responses based on treatment *in utero* could impact risk for naturally acquired infection or morbidity. Our objectives were to evaluate how PZQ treatment among women infected with *S*. *japonicum* might modify the process of *in utero* sensitization and impact a child’s later response to *S*. *japonicum*.

We found that maternal PZQ treatment did not impact the prevalence or intensity of natural *S*. *japonicum* infection among children at age six, although only 37 participants were infected at baseline. These findings agree with a recent study of children born to PZQ-treated mothers with *S*. *mansoni* infection in Uganda [33]. Maternal PZQ also did not impact morbidity outcomes of anemia or hepatic fibrosis, neither in the sample (N = 295) or among the small subset of children with naturally acquired *S*. *japonicum* infection (N = 37). Only two participants were identified with hepatic fibrosis by liver ultrasound, and neither were infected with *S*. *japonicum*. We further evaluated the immunologic responses of these children with particular attention to T-helper cell cytokine profiles; a Th2 response (IL-4, IL-5, IL-13) has more consistently been demonstrated in the context of human resistance to infection, compared to a pro-inflammatory or Th1 response (IFN-γ, IL-1, IL-12, TNF-α). For children without *S*. *japonicum* infection at enrollment, *ex vivo* antigen stimulation of PBMCs revealed that maternal PZQ treatment resulted in lower production of IL-4 against SWAP and higher IL-12 production against SEA compared to placebo. Among children with natural infection at enrollment, we report that PBMCs stimulated with SEA produced higher concentrations of IL-1 in the maternal PZQ treatment group compared to placebo. Overall, maternal PZQ treatment shifted the PBMC antigen-stimulated immune response toward a canonical Th1 profile with reduced IL-4 and increased IL-12 and IL-1 production. Though this shift could theoretically place offspring at increased risk for infection, we did not observe significant differences in the prevalence of infection. It should be noted, however, that only 12.5% of the cohort was infected with *S*. *japonicum* and there was a trend toward increased prevalence of infection among the offspring of PZQ-treated women (16.1%) vs placebo (8.9%). It is reassuring to note, however, that among infected children, there was no significant difference in the intensity of infection comparing offspring of treated and untreated mothers.

With respect to the effect of maternal PZQ treatment on serum cytokines, which may be more proximately related to morbidity risk, we did not find significant differences in any cytokine comparing offspring of treated and untreated mothers. Extending this to the sub-group of children who were infected at the enrollment visit (N = 37), we did not find that maternal PZQ treatment influenced serum cytokines. While maternal PZQ treatment may impact offspring PBMC responses to SWAP and SEA stimulation, serum cytokines during natural infection were not influenced by maternal treatment. The lack of maternal treatment effect on serum cytokines reduces concerns that maternal PZQ treatment may prime children toward a Th1 immune response upon natural infection which could, in turn, promote morbidities such as anemia of inflammation and undernutrition.

Turning to the influence of infection on immune responses, serum TNF-RI was significantly higher among cases of *S*. *japonicum* compared to children who were uninfected. Similar results were reported in the context of *S*. *mansoni* [37]. TNF-RI is a receptor for the pro-inflammatory cytokine TNF-α, which is associated with chronic human schistosomiasis and contributes to granuloma formation [38–41]. TNF-RI is associated with the development liver fibrosis in cases of schistosomiasis [6,42], however, younger children are more likely to experience nutritional morbidities associated with circulating pro-inflammatory cytokines IL-1, IL-6 and TNF-α [43], as clinically relevant fibrosis can take 5–15 years to develop [44].

Among infected children, we did not observe higher concentrations of circulating cytokines typically associated with protective immunity, namely IL-4 or IL-5. Protective immunity develops over the course of adolescence, with repeated and prolonged exposure to *S*. *japonicum* antigens. However, following treatment among children with natural infection in this cohort, PBMC stimulation in response to SWAP and SEA resulted in significant increases in the production of IL-4, IL-5, IL-10, and IL-13 compared to uninfected children. While all the children would have been exposed to *S*. *japonicum* antigens *in utero*, natural infection treatment was a greater driver of potentially protective Th2 responses than *in utero* sensitization.

We acknowledge several limitations to these studies. First, only a small proportion of children were infected at age six. This may be due to receipt of PZQ during MDA campaigns between the ages of five and six. The small sample of natural infections may have impacted our power to detect differences in the effect of maternal PZQ treatment on *S*. *japonicum* infection risk and intensity of infection. With respect to maternal PZQ treatment, there is temporal variability pertaining to both the gestational timing of treatment and the extent and duration of *in utero* sensitization. This cohort was treated relatively early in gestation at 12–16 weeks, which may have reduced *in utero* priming. Variability in MDA treatment in the year before enrollment at age six is another limitation. Though we stratified analyses based on infection at enrollment, it is possible that some of the children who were not infected at enrollment had nonetheless experienced a natural infection previously that was treated during MDA campaigns. MDA of PZQ is recommended for The Philippines, however these programs were interrupted due to Hurricane Haiyan during a time when many of the children were five to six years of age.

We report that maternal PZQ treatment for *S*. *japonicum* did not have a significant impact on their offspring’s likelihood of infection or serum cytokine levels. This report reduces the concern that maternal PZQ treatment during pregnancy exposes the fetus to transplacentally trafficked schistosome antigens and a potentially harmful Th1 response profile, which could theoretically increase risk for infection and morbidity. This provides further reassurance supporting the safe use of PZQ during pregnancy.

## Supporting information

S1 STROBE Statement Checklist of items that should be included in reports of cohort studies. (DOC)
